# RATINGS OF PERCEIVED EXERTION: PREDICTING MOBILITY DISABILITY AND RESPONSE TO PHYSICAL ACTIVITY IN OLDER ADULTS

**DOI:** 10.1093/geroni/igz038.3512

**Published:** 2019-11-08

**Authors:** Erta Cenko, Thomas M Gill, Nancy W Glynn, Marco Pahor, Peihua Qiu, Vincenzo Valiani, Lu You, Todd M Manini

**Affiliations:** 1 University of Florida, Gainesville, Florida, United States; 2 Yale School of Medicine, New Haven, Connecticut, United States; 3 University of Pittsburgh, Department of Epidemiology, Pittsburgh, Pennsylvania, United States; 4 University of Bari, Bari, Italy

## Abstract

Ratings of perceived exertion (RPE) during exercise are linked to several physiological indices and are often elevated in older adults. This study evaluated the association between RPE of walking and incident major mobility disability (MMD) as well as response to a physical activity (PA) program. Older adults (n=1633) at-risk for mobility impairment were randomized to a structured PA or health education (HE) program. During a 400m walk, participants rated exertion as “none”, “light”, “somewhat hard” or “hard”. An MMD event was defined as the inability to complete the 400m walk. Transitions between RPE states and an MMD event—when RPE was not collected— were assessed over the follow-up (every 6 months for an average of 2.6 years). Participants rating their exertion as “hard” at baseline 400m walk had nearly 3-fold higher risk of MMD compared with those rating as “light” (HR: 2.61, 95%CI: 2.19-3.11). During follow-up, the PA group was 25% more likely to transition from “light” to “hard” RPE (1.25, 1.05-1.49), but was 27% (0.73, 0.55 – 0.97) less likely to transition from a “hard” RPE to MMD than the HE group. Additionally, the PA group was more likely to transition from an MMD event to a “hard” RPE (2.09, 1.38-3.17) than the HE group (i.e. recovery). Older adults rating “hard” effort during a standardized walk test were at increased risk of MMD. A structured PA program increased transition from light to hard effort, which may reflect greater capacity to perform the test and increased recovery from an MMD event.

